# Low-dose aspirin and survival from lung cancer: a population-based cohort study

**DOI:** 10.1186/s12885-015-1910-9

**Published:** 2015-11-17

**Authors:** Úna C. Mc Menamin, Chris R. Cardwell, Carmel M. Hughes, Liam M. Murray

**Affiliations:** 1 Cancer Epidemiology and Health Services Research Group, Centre for Public Health, Queen’s University Belfast, Belfast, UK; 2 School of Pharmacy, Queen’s University Belfast, Belfast, BT9 7BL UK; 3 Centre of Excellence for Public Health (NI), Centre for Public Health, Queen’s University Belfast, Belfast, UK

**Keywords:** CPRD, Low-dose aspirin, Lung cancer survival, Pharmacoepidemiology

## Abstract

**Background:**

Preclinical evidence suggests that aspirin may inhibit lung cancer progression. In a large cohort of lung cancer patients, we investigated whether low-dose aspirin use was associated with a reduction in the risk of lung cancer-specific mortality.

**Methods:**

We identified lung cancer patients from English cancer registries diagnosed between 1998 to 2009 from the National Cancer Data Repository. Medication usage was obtained from linkages to the UK Clinical Practice Research Datalink and lung cancer-specific deaths were identified from Office of National Statistics mortality data. Hazard ratios (HR) and 95 % confidence intervals (CI) for the association between low-dose aspirin use (before and after diagnosis) and risk of lung cancer-specific mortality were calculated using Cox regression models.

**Results:**

A total of 14,735 lung cancer patients were identified during the study period. In analysis of 3,635 lung cancer patients, there was no suggestion of an association between low-dose aspirin use after diagnosis and cancer-specific mortality (adjusted HR = 0.96, 95 % CI: 0.85, 1.09). Similarly, no association was evident for low-dose aspirin use before diagnosis and cancer-specific mortality (adjusted HR = 1.00, 95 % CI: 0.95, 1.05). Associations were comparable by duration of use and for all-cause mortality.

**Conclusion:**

Overall, we found little evidence of a protective association between low-dose aspirin use and cancer-specific mortality in a large population-based lung cancer cohort.

## Background

It is increasingly recognised that platelets play a critical role in the progression of cancer [1–3]. The use of aspirin, a commonly prescribed anti-platelet agent, after cancer diagnosis has been associated with a reduction in the risk of recurrence or cancer-specific mortality in colorectal [4, 5], breast [6, 7] and prostate [8] cancer cohorts. Partly motivated by these studies, a large phase 3 randomised trial of aspirin as adjunct treatment is soon to commence including patients at these sites [9]. A similar trial in lung cancer patients was planned but not conducted [10]. Accruing preclinical data suggest that aspirin may have anti-cancer properties [11, 12] by suppressing cellular proliferation [13], reducing neo-vascularisation [14] and inhibiting cell migration and the formation of metastases [15, 16]. Few epidemiological studies have examined the impact of aspirin on the progression of lung cancer, despite promising *in vivo* preclinical evidence of relevance to lung cancer [17, 18] and evidence that lung cancer patients previously exposed to low-dose aspirin present with more favourable tumour characteristics [19]. Only one epidemiological study has investigated cancer-specific outcomes in users of aspirin after lung cancer diagnosis, a time period when clinical intervention is possible. In a small cohort of 643 patients diagnosed with stage III non-small cell lung cancer, Wang et al. [20] reported a substantial, albeit non-significant reduction in the risk of distant cancer metastasis in users of aspirin (but not specifically low-dose) during definitive radiotherapy. Other studies have investigated aspirin use and overall survival but these results could reflect mortality from non-cancer causes. A cohort study of 1,765 non-small cell lung cancer patients reported a significant improvement in overall survival among those using aspirin (but not specifically low-dose) pre-operatively [21]. No difference in the rate of overall survival was observed in patients assigned to an anti-inflammatory daily dose of 1000 mg aspirin compared to non-treatment in a small randomised trial of 303 small cell lung cancer patients [22]. These 3 studies provide limited information as they were not population-based [20, 21], did not investigate low-dose aspirin solely and used limited time-points to ascertain drug exposure. Further epidemiological studies of the impact of low-dose aspirin use on lung cancer progression are therefore warranted to inform the conduct of randomised trials of low dose aspirin as adjunct treatment in lung cancer patients.

In a large population-based cohort of cancer-registry confirmed lung cancer patients utilising detailed prescribing records, we aimed to investigate whether low-dose aspirin use, either before and after diagnosis, was associated with a reduced cancer-specific mortality.

## Methods

### Data sources

This study utilised record linkages between the National Cancer Data Repository (NCDR), the United Kingdom (UK) Clinical Practice Research Datalink (CPRD) and the Office of National Statistics (ONS) death registration data. The NCDR contains data on cancer patients diagnosed in England including the date and site of primary cancer diagnoses, as well as information on cancer treatments received. The CPRD is the world’s largest computerised dataset of anonymised longitudinal primary care records covering approximately 7 % of the United Kingdom population. It comprises general practice records of documented high quality [23, 24] containing demographics, clinical diagnoses and prescriptions issued. Date and cause of death was provided by ONS death registrations. The CPRD group obtained ethical approval from a Multicentre Research Ethics Committee (MREC) for purely observational research using data from the database, such as ours. This study obtained approval from the Independent Scientific Advisory Committee (ISAC) of the CPRD, which is responsible for reviewing protocols for scientific quality.

### Study design

Between 1998 and 2009, all patients newly diagnosed with primary lung cancer (International Classification of disease, ICD code C34) were identified from the NCDR. Patients with a previous NCDR cancer diagnosis were excluded, with the exception of in situ neoplasms and non-melanoma skin cancers. Using ONS death registration data, deaths were obtained up until January 2012 and lung cancer specific deaths were identified using an underlying cause of death ICD code C34.

### Exposure data

General practitioner (GP)-recorded aspirin prescriptions, according to the British National Formulary [25], were classified as low if ≤75 mg (0.1 % of prescriptions after diagnosis 25 mg, 92.7 % were 75 mg and 7.3 % were >75 mg). The average quantity of 28 was assumed for approximately 2 % of prescriptions were quantity was missing or incorrect.

### Covariates

Clinical data on tumour histology, and receipt of cancer treatments including surgery, chemotherapy and radiotherapy within 6 months after diagnosis was obtained from the NCDR. Tumour histology was based on cancer registry recorded International Classification of Diseases for Oncology codes (3^rd^ Edition). Data on lifestyle factors including smoking, alcohol and BMI was derived from the closest GP records within 10 years prior to diagnosis. Clinical GP-recorded diagnoses were used to determine comorbidities prior to diagnosis, and comprised those which were included in a recent adaptation of the Charlson Comorbidity index [26]. A measure of deprivation was available from CPRD records based on the 2004 Index of Multiple Deprivation for England which comprises super output area (SOA) level measures of multiple deprivation (based on UK residential postcodes) and is made up of seven SOA level domain indices [27]. Patients were categorised into one of 5 quintiles of deprivation with the first quintile representing the least deprived and the fifth quintile representing the most deprived. Other medications including statins and beta-blockers, were determined from GP-prescription records and included in adjusted analyses due to potential associations with cancer-specific mortality.

### Statistical analysis

#### Statistical analysis for low-dose aspirin use after diagnosis

In the analysis of low-dose aspirin use after diagnosis (regardless of pre-diagnostic low-dose aspirin use), lung cancer patients who died in the first year after diagnosis were excluded (sensitivity analysis was conducted varying this interval) as it is likely that these patients had stage IV disease and it seemed unlikely that short term post-diagnostic drug use would influence such deaths. Patients were therefore followed up from one year after diagnosis until death, end of registration with the general practice, last date of data collection from general practice or end of ONS follow-up. Time dependent Cox regression models were used to produce unadjusted and adjusted hazard ratios (HRs) and 95 % confidence intervals (CIs) for the association between low-dose aspirin use and lung cancer-specific mortality. Low-dose aspirin use was treated as time-varying, with users not considered to be exposed until after a lag of six months following their initial prescription. Other medications including statins and beta-blockers were treated in a similar manner. The use of a lag is recommended [28] and was used to exclude prescriptions in the six months prior to death as these may reflect changes due to end of life care. Medications may be withdrawn from cancer patients in whom death is suspected to be imminent and unlagged time-varying covariate analysis can bias results due to reverse causality [29]. Dose–response relationships were investigated by cumulative number of prescriptions and increasing number of tablets during the exposure period, and analyses were repeated for all-cause mortality. Sub-group analyses were carried out by sex, pre-diagnostic low-dose aspirin use, histology and surgery within 6 months after diagnosis. Tests for interactions were performed for each sub-group analysis. Separate sensitivity analyses were conducted by: increasing the lag from 6 months to 1 year (thereby excluding prescriptions in the year prior to death); only excluding those who died within the first six months after diagnosis (thereby including more of the cohort); and additionally adjusting for smoking, BMI and histological sub-type. A simplified analysis was conducted assessing the influence of low-dose aspirin use versus non-use in the first year after lung cancer diagnosis among patients who survived at least one year after diagnosis. In order to verify the robustness of results (i.e. if the findings are similar to the main analysis it would suggest that our results are robust), the entire cohort was converted to case–control data to carry out a nested case–control analysis using conditional logistic regression. Cases were patients that died due to lung cancer and were matched on sex, age (in 5 year bands) and year of diagnosis (in 2 year bands) to five risk-set controls that lived at least as long after their lung cancer diagnosis, thereby eliminating immortal time bias [30]. Odds ratios (ORs) and 95 % confidence intervals (CIs) were produced using conditional logistic regression to examine the association between low-dose aspirin use and lung cancer-specific mortality.

### Statistical analysis for low-dose aspirin use before diagnosis

In the analyses of low-dose aspirin use before lung cancer diagnosis, follow-up began from diagnosis until death or censoring (as described earlier). Patients who died in the first year after diagnosis were not excluded. Cox regression models were used to calculate unadjusted and adjusted HRs and 95 % CIs based upon prescriptions in the year prior to diagnosis, among patients with at least 1 year of CPRD records prior to diagnosis. To prevent over-adjustment in the analysis of pre-diagnosis low-dose aspirin use, adjustments were only made for potential confounders recorded prior to cancer diagnosis [31, 32] (statin and beta-blocker use were also based upon prescriptions in the year prior to diagnosis). Analyses were conducted by cumulative number of low-dose aspirin prescriptions and increasing number of tablets within the exposure period, and repeated for all-cause mortality. Sub-group analyses were carried out by sex and sensitivity analyses included additional adjustment for smoking and BMI prior to diagnosis and extending the pre-diagnostic exposure period from 2 years to 6 months prior to diagnosis (among patients with at least 2 years of records prior to diagnosis).

## Results

### Patient cohort

A total of 14,735 lung cancer patients with linked CPRD data were identified from the NCDR. The analysis of aspirin use after diagnosis included 3,635 patients after excluding 11,100 patients with less than 1 year of follow-up (10,295 of whom had died). The analysis of aspirin use before diagnosis included 13,433 patients, after excluding 1,302 patients with less than 1 year of CPRD records prior to diagnosis. In the analysis of aspirin use after diagnosis average follow-up was 3 years (maximum 14 years) and in the analysis of aspirin use before diagnosis, average follow-up was 1 year (maximum 14 years).

### Patient characteristics

Table 1 lists patient characteristics by low-dose aspirin use. Users of low-dose aspirin either before or after diagnosis were more likely to be diagnosed more recently, be older, be male and be overweight or obese prior to cancer diagnosis. The majority of comorbidities were also more common in users of aspirin (particularly cerebrovascular disease, diabetes and myocardial infarction), in addition to the use of statins and beta-blockers. Low-dose aspirin users after diagnosis were less likely to undergo chemotherapy. Other patient characteristics were not as strongly associated with the use of low-dose aspirin.

**Table 1:** Characteristics of lung cancer patients by low-dose aspirin use

Characteristics	Total study population	Low-dose aspirin use in year prior to diagnosis^a^		Low-dose aspirin use after diagnosis^b^	
		User n (%)	Non-user (%)	Ever n (%)	Never (%)
	*(n = 14,735)*	*(n = 13,433)*		*(n = 3,635)*	
Year of diagnosis: 1998–2000	2,797 (19)	459 (12)	1,850 (19)	175 (15)	463 (19)
2001–2003	3,708 (25)	831 (22)	2,524 (26)	280 (24)	603 (24)
2004–2006	4,025 (27)	1,175 (30)	2,603 (27)	322 (28)	647 (26)
2007–2009	4,205 (29)	1,404 (36)	2,587 (27)	384 (33)	761 (31)
Age at diagnosis: < 50	492 (3)	11 (0)	410 (4)	10 (1)	163 (7)
50–59	1,780 (12)	171 (4)	1,425 (15)	97 (8)	475 (19)
60–69	3,912 (27)	916 (24)	2,660 (28)	362 (31)	804 (33)
70–79	5,347 (36)	1,681 (44)	3,231 (34)	478 (31)	775 (31)
80–89	2,898 (20)	974 (25)	1,677 (18)	199 (17)	242 (10)
≥90	306 (2)	116 (3)	161 (2)	15 (1)	15 (1)
Gender: Males	8,701 (59)	1,581 (63)	6,360 (58)	748 (64)	1,357 (55)
Treatment within 6 months of cancer diagnosis					
Surgery^c^	1,324 (12)	329 (11)	895 (12)	305 (34)	567 (30)
Chemotherapy	3,287 (22)	709 (18)	2,324 (24)	309 (27)	950 (38)
Radiotherapy	4,668 (32)	1,129 (29)	3,100 (32)	389 (34)	989 (40)
Histology: Non-small cell	8,224 (56)	2,066 (53)	5,478 (57)	822 (71)	1,793 (73)
Small cell	1,828 (12)	458 (12)	1,218 (13)	121 (10)	303 (12)
Missing	4,683 (32)	1,345 (35)	2,868 (30)	218 (19)	378 (15)
Smoking status prior to cancer diagnosis					
Non-smoker	1,907 (13)	543 (14)	1,188 (12)	148 (13)	339 (14)
Ex-smoker	5,214 (35)	1,754 (45)	3,111 (33)	541 (47)	852 (34)
Current smoker	5,961 (41)	1,329 (34)	4,138 (43)	393 (34)	1,042 (42)
Missing	1,653 (11)	243 (6)	1,127 (12)	79 (7)	241 (10)
Alcohol consumption prior to diagnosis					
Never	2,311 (16)	727 (19)	1,385 (15)	190 (16)	342 (14)
Ever	9,707 (66)	2,678 (69)	6,337 (66)	841 (72)	1,712 (69)
Missing	2,717 (18)	464 (12)	1,842 (19)	130 (11)	420 (17)
BMI (kg/m^2^) prior to diagnosis: mean (sd)					
Underweight (<18.5)	735 (5)	175 (5)	487 (5)	38 (3)	100 (4)
Normal (18.5 to 25)	5,325 (36)	1,379 (36)	3,543 (37)	401 (35)	943 (38)
Overweight (25–30)	3,916 (27)	1,199 (31)	2,444 (26)	388 (33)	695 (28)
Obese (>30)	1,702 (12)	602 (16)	1,002 (11)	196 (17)	287 (12)
Missing	3,057 (21)	514 (13)	2,088 (22)	138 (12)	449 (18)
Deprivation fifth: 1^st^ (least deprived)	2,583 (18)	650 (17)	1,696 (18)	206 (18)	481 (19)
2^nd^	2,822 (19)	754 (20)	1,833 (19)	236 (20)	451 (18)
3^rd^	2,975 (20)	778 (20)	1,954 (20)	232 (20)	492 (20)
4^th^	3,235 (22)	815 (21)	2,102 (22)	250 (22)	556 (23)
5^th^ (most deprived)	3,062 (21)	859 (22)	1,947 (20)	233 (20)	489 (20)
Missing	58 (0)	13 (0)	32 (0)	4 (0)	5 (0)
Comorbidity prior to cancer diagnosis					
Cerebrovascular disease	1,.325 (9)	746 (19)	498 (5)	147 (13)	95 (4)
Chronic pulmonary disease	3,619 (25)	1,054 (27)	2,341 (25)	307 (26)	621 (25)
Congestive heart disease	954 (7)	413 (11)	483 (5)	83 (7)	76 (3)
Diabetes	1,552 (11)	758 (20)	698 (7)	194 (17)	141 (6)
Myocardial infarction	1,262 (9)	866 (22)	316 (3)	223 (19)	67 (3)
Peptic ulcer disease	1,087 (7)	264 (7)	745 (8)	65 (6)	182 (7)
Peripheral vascular disease	1,422 (10)	780 (20)	576 (6)	200 (17)	127 (5)
Renal disease	864 (6)	441 (11)	397 (4)	97 (8)	99 (4)
Other medication use after diagnosis					
Statin use^d^	4,801 (33)	2,103 (54)	1,193 (13)	705 (61)	410 (17)
Beta-blocker use^d^	3,823 (26)	1,143 (30)	1,005 (11)	416 (36)	272 (11)

^a^Analysis includes lung cancer patients who have more than 1 year of records prior to diagnosis

^b^Post-diagnostic aspirin use (regardless of pre-diagnostic aspirin use), among lung cancer patients who lived more than 1 year after diagnosis

^c^Excluding cancer patients from Thames Registry as surgery information not available

^d^Statin and beta-blocker use ever after diagnosis for low-dose aspirin use after diagnosis columns, statins and beta-blocker use in year prior to diagnosis for low-dose aspirin use in year prior to diagnosis columns, statins and beta-blocker use either before or after diagnosis in total study population column

### Association between low-dose aspirin use after diagnosis and survival

There was no evidence of an association between low-dose aspirin use after diagnosis and lung cancer-specific mortality (HR = 0.96, 95 % CI: 0.87, 1.05), as shown in Table 2. No dose–response relationship was evident by increasing prescriptions of low-dose aspirin, or by tablets. Similarly, no difference in the rate of all-cause mortality was observed between users of low-dose aspirin and non-users, Table 2. Adjustment for potential confounders including cancer treatments and comorbidities did not materially alter risk estimates. In sub-group analyses, associations between low-dose aspirin use and cancer-specific mortality did not differ by sex, pre-diagnostic low-dose aspirin use or surgical treatment, see Table 3. There was a suggestion of a small, although not statistically significant, reduction in the risk of cancer-specific mortality in patients diagnosed with small cell lung cancer using low-dose aspirin after diagnosis (adjusted HR = 0.72, 95 % CI: 0.52, 1.01; *P* for interaction = 0.034). Results from sensitivity analyses were comparable to that of the main analysis, Table 3.

**Table 2:** Association between low-dose aspirin usage after cancer diagnosis and cancer-specific and all-cause mortality in lung cancer patients

Medication usage after diagnosis	Cancer-specific deaths	All-cause mortality	All patients	Person years	Cancer-specific mortality						All-cause mortality					
					Unadjusted		*P*	Adjusted^a^		*P*	Unadjusted		*P*	Adjusted^a^		*P*
					HR	95 % CI		HR	95 % CI		HR	95 % CI		HR	95 % CI	
*Number of patients*					*[n = 3,635]*			*[n = 2,791]*			*[n = 3,635]*			*[n = 2,791]*		
Aspirin non-user	1,609	1,855	2,474	4,481	1.00	Referent		1.00	Referent		1.00	Referent		1.00	Referent	
Aspirin user^b^	638	795	1,161	2,264	0.96	0.87, 1.05	0.36	0.96	0.85, 1.09	0.55	1.00	0.92, 1.09	0.92	0.94	0.84, 1.05	0.28
Aspirin non-user	1,609	1,855	2,474	4,481	1.00	Referent		1.00	Referent		1.00	Referent		1.00	Referent	
Aspirin user 1 to 11 prescriptions^c^	440	521	670	1,189	0.94	0.85, 1.04	0.25	0.95	0.83, 1.08	0.42	0.97	0.88, 1.07	0.60	0.93	0.83, 1.05	0.24
Aspirin user ≥ 12 prescriptions^c^	198	274	491	1,075	1.00	0.86, 1.17	0.96	1.01	0.84, 1.23	0.89	1.07	0.94, 1.23	0.29	0.97	0.82, 1.14	0.69
Aspirin non-user	1,609	1,855	2,474	4,481	1.00	Referent		1.00	Referent		1.00	Referent		1.00	Referent	
Aspirin user 1–365 tablets^c^	353	422	541	926	0.91	0.81, 1.02	0.12	0.92	0.80, 1.06	0.27	0.96	0.86, 1.07	0.44	0.92	0.81, 1.05	0.21
Aspirin user ≥366 tablets^c^	285	373	620	1,338	1.03	0.90, 1.17	0.69	1.04	0.88, 1.23	0.67	1.07	0.95, 1.20	0.28	0.97	0.84, 1.13	0.72

^a^Adjusted for year of diagnosis, age at diagnosis, gender, radiotherapy within 6 months, chemotherapy within 6 months, surgery within 6 months, comorbidities (prior to diagnosis, including cerebrovascular disease, chronic pulmonary disease, congestive heart disease, diabetes, myocardial infarction, peptic ulcer disease, peripheral vascular disease, renal disease), other medication use (after diagnosis, as time varying covariates, specifically statins and beta-blockers) and deprivation (in fifths)

^b^Medication use modelled as a time varying covariate with an individual considered a non-user prior to 6 months after first medication usage and a user after this time, excludes deaths in the year after cancer diagnosis

^c^Medication use modelled as a time varying covariate with an individual considered a non-user prior to 6 months after first medication usage, a user of 0 to 12 prescriptions from 6 months after first prescription to 6 months after 12^th^ prescription (or 365 tablets) and a greater user after this time, excludes deaths in the year after cancer diagnosis

**Table 3:** Sensitivity analyses for association between low-dose aspirin use and cancer-specific mortality in lung cancer patients

	Cancer-specific deaths	All patients	Person years	User versus non-user			User versus non-user			
				Unadjusted		*P*	Adjusted HR^a^		*P*	*P* for interaction
				HR^a^	95 % CI		HR^a^	95 % CI		
Main analysis: Aspirin user versus non-user after diagnosis	2,247	3,635	6,745	0.96	0.87, 1.05	0.36	0.96	0.85, 1.09	0.55	
Sub group analyses: Aspirin user versus non-user after diagnosis, restricted to:										
Males	1,312	2,105	3,750	1.00	0.89, 1.12	0.97	1.05	0.90, 1.22	0.54	0.12
Females	935	1,530	2,995	0.87	0.75, 1.02	0.08	0.81	0.66, 0.99	0.05	
Pre-diagnosis aspirin non-users^b^	1,515	2,446	4,578	0.89	0.74, 1.07	0.21	0.91	0.73, 1.14	0.42	0.48
Pre-diagnosis aspirin users^b^	554	908	1,601	0.92	0.72, 1.16	0.47	1.02	0.77, 1.35	0.90	
Small cell lung cancer	328	424	592	0.84	0.66, 1.09	0.19	0.72	0.52, 1.01	0.05	0.03
Non-small cell lung cancer	1,523	2,615	5,355	0.95	0.85, 1.06	0.35	1.00	0.86, 1.16	0.99	
Surgically treated	305	872	2,714	0.99	0.78, 1.27	0.96	0.95	0.71,1.28	0.74	0.39
Non-surgically treated	820	1,708	4,216	0.94	0.80, 1.10	0.42	0.89	0.74, 1.07	0.22	
Sensitivity analyses: Aspirin user versus non-user after diagnosis										
Increasing lag to 1 year	2,247	3,635	6,745	0.97	0.88, 1.07	0.56	0.97	0.85, 1.10	0.60	
Excluding patients who died within the first 6 months after diagnosis	4,440	6,158	9,101	0.96	0.90, 1.03	0.28	0.95	0.87–1.04	0.30	
Additionally adjusting for smoking prior to diagnosis	2037	3,315	6,074	0.95	0.86, 1.04	0.28	0.95	0.84, 1.08	0.41	
Additionally adjusting for BMI prior to diagnosis	1,849	3,048	5,640	0.93	0.84, 1.03	0.15	0.95	0.84, 1.09	0.47	
Additionally adjusting for small cell/non-small cell	1,851	3,039	5,947	0.92	0.83, 1.03	0.14	0.92	0.80, 1.05	0.23	
Based upon first year after diagnosis^c^	1,728	2,791	5,223	0.93	0.87, 1.05	0.33	0.99	0.88, 1.13	0.91	
Nested case–control analysis^d,e^	2,247			0.93	0.85, 1.03	0.19	1.00	0.86, 1.16	0.97	

^a^Except where otherwise stated, all analyses of post-diagnostic aspirin use adjusted for year of diagnosis, age at diagnosis, gender, surgery within 6 months of diagnosis, radiotherapy within 6 months, chemotherapy within 6 months, comorbidities (prior to diagnosis, including cerebrovascular disease, chronic pulmonary disease, congestive heart disease, diabetes, myocardial infarction, peptic ulcer disease, peripheral vascular disease, renal disease), other medication use (after diagnosis, as time varying covariates, specifically statins and beta-blockers) and deprivation (in fifths)

^b^Based upon aspirin use in the year prior to diagnosis, restricted to individuals with 1 year of records prior to lung cancer diagnosis

^c^Simplified analysis, not requiring time varying covariate use, comparing aspirin users to aspirin non-users in the first year after diagnosis in individuals living more than 1 year after cancer diagnosis, adjusted for all confounders in^a^ but other medication use also restricted to first year after diagnosis

^d^Unadjusted OR estimate and 95 % CIs based on 28 % (623/2,247) of cancer-specific deaths using aspirin compared with 31 % (3,254/10,603) of risk-set controls (not dying from cancer)

^e^Adjusted OR estimate and 95%CIs, matched on age at diagnosis, year of diagnosis, gender and adjusted for all other confounders in^a^

### Association between low-dose aspirin use before diagnosis and survival

Overall, no association between aspirin use prior to diagnosis and lung cancer-specific mortality was observed (adjusted HR = 1.00, 95 % CI: 0.95, 1.05) and no dose–response relationship was apparent in analyses by increasing prescriptions or tablets, Table 4. Similar associations were observed across sub-group and sensitivity analyses; for example, after additional adjustment for smoking and BMI (Table 5).

**Table 4:** Association between low-dose aspirin usage in the year prior to diagnosis and cancer-specific and all-cause mortality in lung cancer patients

Medication usage after diagnosis	Cancer-specific deaths	All-cause mortality	All patients	Person years	Cancer-specific mortality						All-cause mortality					
					Unadjusted		*P*	Adjusted^a^		*P*	Unadjusted		*P*	Adjusted^a^		*P*
					HR	95 % CI		HR	95 % CI		HR	95 % CI		HR	95 % CI	
*Number of patients*					*[n = 13,433]*			*[n = 13,388]*			*[n = 13,433]*			*[n = 13,388]*		
Aspirin non-user	7,577	8,369	9,564	9,154	1.00	Referent		1.00	Referent		1.00	Referent		1.00	Referent	
Aspirin user	3,055	3,468	3,869	3,331	1.07	1.02, 1.11	<0.01	1.00	0.95, 1.05	0.91	1.10	1.05, 1.14	<0.001	1.01	0.96, 1.06	0.77
Aspirin non-user	7,577	8,369	9,564	9,154	1.00	Referent		1.00	Referent		1.00	1.00		1.00	Referent	
Aspirin user 1 to 11 prescriptions	2,367	2,690	2,998	2,608	1.06	1.01, 1.11	0.02	0.99	0.94, 1.04	0.75	1.09	1.04, 1.14	<0.001	1.00	0.95, 1.05	0.90
Aspirin user ≥ 12 prescriptions	688	778	871	723	1.09	1.01, 1.18	0.03	1.02	0.94, 1.11	0.64	1.11	1.04, 1.20	<0.01	1.02	0.95, 1.11	0.57
Aspirin non-user	7,577	8,369	9,564	9,154	1.00	Referent		1.00	Referent		1.00	1.00		1.00	Referent	
Aspirin user 1–365 tablets	2,214	2,508	2,804	2,436	1.06	1.01, 1.12	0.01	1.00	0.95, 1.06	0.97	1.09	1.04, 1.14	<0.001	1.01	0.96, 1.06	0.70
Aspirin user ≥366 tablets	841	960	1,065	895	1.07	1.00, 1.15	0.06	0.98	0.91, 1.06	0.70	1.11	1.04, 1.18	<0.01	1.00	0.93, 1.07	0.97

^a^Adjusted for year of diagnosis, age at diagnosis, gender, comorbidities (prior to diagnosis, including cerebrovascular disease, chronic pulmonary disease, congestive heart disease, diabetes, myocardial infarction, peptic ulcer disease, peripheral vascular disease, renal disease), other medication use (in year prior to diagnosis, specifically statins and beta-blockers) and deprivation (in fifths)

**Table 5:** Sensitivity analyses for association between low-dose aspirin use and cancer-specific mortality in lung cancer patients

	Cancer-specific deaths	All patients	Person years	Unadjusted		*P*	Adjusted^a^		*P*
				HR	95 % CI		HR	95 % CI	
Main analysis: Pre-diagnostic aspirin use^b^	10,632	13,433	12,485	1.07	1.02, 1.11	<0.01	1.00	0.95, 1.05	0.91
Subgroup analyses									
Male	6,298	7,941	7,103	1.02	0.97, 1.08	0.44	0.98	0.91–1.04	0.46
Female	4,334	5,492	5,381	1.13	1.06–1.21	<0.001	1.03	0.95–1.11	0.53
Sensitivity analyses									
Smoking prior to diagnosis available (and adjusted for)	9,560	12,063	11,336	1.07	1.03–1.12	<0.01	1.00	0.95–1.05	0.93
BMI prior to diagnosis available (and adjusted for)	8,562	10,831	10,448	1.07	1.02–1.12	<0.01	0.99	0.94–1.05	0.73
Aspirin use between 2 years and 6 months prior to diagnosis^c^	9,746	12,295	11,418	1.06	1.01–1.11	0.01	0.99	0.94–1.05	0.76

^a^Except where otherwise stated, adjusted for year of diagnosis, age at diagnosis, gender, comorbidities (prior to diagnosis, including cerebrovascular disease, chronic pulmonary disease, congestive heart disease, diabetes, myocardial infarction, peptic ulcer disease, peripheral vascular disease, renal disease), other medication use (in year prior to diagnosis, specifically statins and beta-blockers) and deprivation (in fifths)

^b^Based upon use in the year prior to diagnosis, restricted to individuals with 1 year of records prior to lung cancer diagnosis

^c^Restricted to individuals with 2 years of records prior to diagnosis, removing prescriptions in the 6 months prior to lung cancer diagnosis as these could reflect increased medical care due to early symptoms

## Discussion

In this population-based study, we did not find evidence of a protective association between low-dose aspirin use and cancer-specific or all-cause mortality in a large cohort of lung cancer patients. Only one previous study has assessed the impact of aspirin on lung cancer-specific outcomes. An American study of 643 non-small cell lung cancer patients conducted by Wang et al. [20] observed a substantial non-significant decrease in the risk of distant metastasis in users of aspirin (but not specifically low-dose) after diagnosis (HR = 0.75, 95 % CI: 0.55–1.03). Inconsistencies between the findings of this study and ours could reflect differences in the methodologies employed. Their study was hospital-based, used a different outcome (distant metastasis), as well as a different method to ascertain aspirin exposure (based upon patient recall during the receipt of definitive radiotherapy). A further study by Fontaine et al. [21], based in the UK, observed a significant reduction in all-cause mortality among pre-operative users of aspirin (HR = 0.84, *P* = 0.05). The authors hypothesised that the observed benefit in all-cause mortality may be due to an improvement in cardiovascular-related mortality, as the reduction in risk was most evident after 3 years [21]. In contrast, we found no evidence of a protective association between low-dose aspirin use and all-cause mortality. A meta-analysis of randomised controlled trials of low-dose aspirin (in patients with increased cardiovascular risk) observed reduced mortality due to lung cancer but this largely reflected lung cancer incidence as patients were cancer-free at randomisation [33]. Our study only investigated low-dose aspirin, hence we cannot rule out a possible benefit of high-dose cycloxygenase (COX)-2 inhibitory aspirin. A previous study, although relatively small and based on patient report, did not observe an association between pre-diagnostic high- or low-dose aspirin and lung cancer survival [34]. Furthermore, our study contained relatively few very long term low-dose aspirin users (i.e. more than 5 years) and therefore it is difficult to comment on the effect of very long term aspirin use.

In sensitivity analyses, we observed a non-significant 28 % reduction in risk of lung cancer-specific mortality with post-diagnostic aspirin use in patients diagnosed with small cell lung cancer. Caution however is required in the interpretation of this finding as this was not an a priori defined subgroup analysis and was based on substantially smaller numbers compared to the main analysis (328 versus 2,247 deaths, respectively).

Our study had a number of strengths and limitations. This is the first study to evaluate the impact of low-dose aspirin use and lung cancer-specific mortality. The cohort was identified from the NCDR, a large population-based resource which allowed for robust verification of cancer diagnoses. Similarly, deaths could be confirmed using ONS. Some misclassification of deaths could have occurred but evidence from methodological comparative studies suggest that risk estimates are unlikely to be greatly affected [35]. The use of high-quality [22] GP-recorded prescriptions allowed for detailed investigation of temporal associations and eliminates potential for recall bias. Over-the-counter usage of low-dose aspirin is possible but previous investigation within the General Practice Research Database found that the majority of chronic aspirin use was captured by prescription records [36]. Furthermore, valid treatment risk estimates have been previously demonstrated when there is potential for over-the-counter medication usage [37]. Drug compliance was unknown in this study but similar results were produced in analysis of multiple prescriptions, in which drug adherence may be more likely. Although we adjusted for a range of potential confounding factors, residual confounding caused by unrecorded or incomplete data cannot be ruled out. More specifically, we were unable to adjust for cancer stage in our analyses; however, as stage may lie on the causal pathway, such adjustments may not be appropriate for the analysis of low-dose aspirin use before diagnosis. Finally, although follow-up time after diagnosis was up to 14 years in both analysis of post-diagnostic and pre-diagnostic low-dose aspirin use, the average follow-up time in each analysis was substantially shorter reflecting poor survival after lung cancer diagnosis (3 years and 1 year, respectively).

## Conclusions

In this population-based study, low-dose aspirin use was not associated with an improvement in cancer survival in a large cohort of cancer-registry confirmed lung cancer patients.

## Consent statement

Informed patient consent was not required for this study.

## Abbreviations

BMI: body mass index
CIs: confidence intervals
CPRD: clinical practice research datalink
GP: general practitioner
HR: hazard ratio
ICD: International classification of diseases
NCDR: National cancer data repository
ONS: Office of national statistics
OR: Odds ratio
UK: United Kingdom
